# Light-Engineering Technology for Enhancing Plant Disease Resistance

**DOI:** 10.3389/fpls.2021.805614

**Published:** 2022-02-17

**Authors:** Duan Wang, Bishnu Dawadi, Jing Qu, Jian Ye

**Affiliations:** ^1^State Key Laboratory of Plant Genomics, Institute of Microbiology, Chinese Academy of Sciences, Beijing, China; ^2^CAS Center for Excellence in Biotic Interactions, University of Chinese Academy of Sciences, Beijing, China; ^3^Beijing Institutes of Life Science, Chinese Academy of Sciences, Beijing, China

**Keywords:** light, plant defense, insect-borne disease, tripartite interaction, LEDs

## Abstract

Insect vector-borne diseases are a major constraint to a wide variety of crops. Plants integrate environmental light and internal signalings to defend dual stresses both from the vector insects and vector-transmitted pathogens. In this review, we highlight a studies that demonstrate how light regulates plants deploying mechanisms against vector-borne diseases. Four major host defensive pathways involved in the host defense network against multiple biotic stresses are reviewed: innate immunity, phytohormone signaling, RNA interference, and protein degradation. The potential with light-engineering technology with light emitting diodes (LEDs) and genome engineering technology for fine-tuning crop defense and yield are also discussed.

## Introduction

Global warming has driven the emergence and reemergence of insect vector-borne plant diseases soaring and causes a huge loss in agricultural production. These microbial pathogens and parasites, especially viruses and bacteria, are transmitted by arthropod vectors. Efforts to control these diseases have been emphasized on the use of chemical pesticides. Pesticide abuse has resulted in pesticide resistance on these extremely polyphagous arthropod species, *via* either physiological, biochemical, or behavioral mechanisms. Especially for viruses, approximately 80% of 1,480 known plant viruses are arthropod vector transmitted and cause billions of dollars loss annually (Ye et al., 2021). The outspreading diseases caused by insect-transmitted plant viruses in the past decades have been mainly driven by planthoppers, whiteflies, aphids, and thrips (Dáder et al., 2015; Wu and Ye, 2020). Besides chemical measures, both the biological and physical strategies, such as light quality and quantity, have recently been highlighted for controlling plant viral diseases (Montes and Pagán, 2019; Zhai et al., 2020; Gallé et al., 2021).

Numerous pieces of evidence have demonstrated that different kinds of light function as antimicrobial and antiviral therapies against human bacterial and viral diseases. Violet/blue light accounts for the Nobel Prize in 1903 given to Niels Ryberg Finsen for the successful treatment of tuberculosis caused by *Mycobacterium tuberculosis*. In a similar way, the potential reduction of phytopathogenic infections in plants could be brought out by the ubiquity of inexpensive light emitting lasers or light emitting diodes (LEDs), which makes it easier to develop safe and low-cost devices. Balancing the effects of light spectrum on crop growth and crop protection is, therefore, required for optimal crop production and quality. The function of light in regulating plant abiotic stress responses, such as temperature responses and drought resistance, to maintain the normal growth and development of plants has been widely reported (D’Amico-Damião and Carvalho, 2018; Szalai et al., 2018; Roeber et al., 2021; Wang et al., 2021a). In this review, we address fundamental aspects of plant responses to supplemental light of specific wavelengths from the perspective of developing greenhouse crop production. We focus on the herbivorous insect as biotic stress because their behavior, as well as the behavior of their natural enemies, may also be affected by supplemental light. The effects of additional far-red (FR), red, blue, and UV components of the light spectrum on ambient greenhouse conditions fundamentally change host plant traits including disease resistance. Although we focus on the effects of supplemental LED light of specific wavelengths, we note that similar results may be obtained with other artificial light sources that also provide specific wavelengths.

Instead of directly killing pathogens, environmental light regulates plant resistance to defend against the invasion of pathogens. Plants have evolved multilayered defense mechanisms including innate immunity, hormone signaling, autophagy and/or 26S proteasome-mediated protein degradation, and RNA interference (RNAi) in plant-pathogen interactions. Insect-borne pathogens are highly dependent on their insect vectors during their natural transmission cycle. Tripartite interaction study among plant–pathogen–insect is more complicated compared with bipartite interactions including plant–pathogen and plant-insect. Understanding plant defensive responses upon multiple stresses simultaneously are of greater significance for developing efficient disease-control strategies. In this review, we mainly summarized the recent advancements in plant defensive pathways, mainly containing plant innate immune response, hormone signaling pathways, RNAi, and protein degradation pathways, combined with the regulation of light signaling toward these resistance pathways.

## Light Signaling Pathway in Plants

Light is indispensible for the growth and stress responses in the whole life of plants since it is the only energy source in a form of electromagnetic radiation from solar. Likewise, light has properties of both the waves and particles and is able to induce DNA damage and other plant stress responses. Photosynthesis in chloroplasts, which is believed to be descended from a prokaryotic ancestor, is one main way for plants to perceive light signals. Photosynthetic processes in plants mainly absorb a photon and use visible light with wavelengths of 400–700 nm. Not all the wavelengths of lights have equal energy and the energy content of light is inversely proportional to its wavelength. Excess and fluctuating light results in reactive oxygen species (ROS) accumulation around photosystems II and I, respectively. ROS accumulation leads to broad-spectrum tolerance against both the abiotic and biotic stresses (Kangasjärvi et al., 2014; Liu et al., 2019; Roeber et al., 2021). Besides photosynthesis, plants sense light in a second way with a series of photoreceptors located in the cytoplasm and nucleus as well as their downstream factors that function in light sensing and signal transduction. Plant photoreceptor-mediated light signalings play fundamental roles in plant growth and defensive responses.

At least five classes of photoreceptors sense unique light wavelengths (Paik and Huq, 2019; Roeber et al., 2021). Phytochromes (PHYS), which mainly sense red and FR light (600–750 nm), contain phyA-phyE five receptors in *Arabidopsis thaliana*. Cryptochromes (CRYs), encoded by *CRY1* and *CRY2*, sense blue, green, and UVA light (320–500 nm) (Folta and Maruhnich, 2007; Liu et al., 2016). Phototropins (PHOTs) (known as PHOT1 and PHOT2) as well as ZEITLUPE (ZTL)/FLAVIN-BINDING, KELCH REPEAT, F-BOX 1 (FKF1)/LOV KELCH PROTEIN2 (LKP2) are illustrated as blue-light receptors (Christie, 2007; Zoltowski and Imaizumi, 2014). The last type of recognized photoreceptor is UV RESISTANCE LOCUS 8 (UVR8), which senses UVB radiation (280–320 nm) (Demkura and Ballaré, 2012; Heijde and Ulm, 2012; Liang et al., 2019). Once the various light signals are detected by these receptors respectively, plants will initiate the downstream responses to regulate their growth, development, and immunity. These photoreceptors regulate either the core factors of the common pathway such as CONSTITUTIVE PHOTOMORPHOGENIC 1/SUPPRESSOR OF PHYA1 (COP1/SPA1) complex or distinctive branches of their sensed signals to mediate light perception responses of plants. COP1/SPA1 complex is an E3 ubiquitin ligase and is usually considered as a negative regulator of photoreceptors regulated light responses which is by degradation of numerous positive transcription factors of light signaling pathways such as ELONGATED HYPOCOTYL 5 (HY5), LONG AFTER FAR-RED LIGHT 1 (LAF1), LONG HYPOCOTYL IN FAR-RED 1 (HFR1), and so on (Lau and Deng, 2012).

Phytochromes (such as phyB) can directly interact with the COP1/SPA complex and interfere with its function in HY5 degradation, which ultimately contributes to photomorphogenesis (Hoang et al., 2019; Li et al., 2020). Except for this indirect regulation of negative factors for photomorphogenesis, phytochromes are also able to directly interact with phytochrome-interacting factors (PIFs) and inhibit their roles in photomorphogenesis repression by phosphorylation and polyubiquitylation-mediated degradation pathway (Park et al., 2004; Shen et al., 2005, 2008; Al-Sady et al., 2006). Through indirect and direct interactions with these master factors in the light pathway, phytochromes regulate the whole living life of plants from seed germination, photomorphogenesis to flowering time, as well as shade avoidance, circadian clock, gravitropism, and even more importantly, the defense responses (Correll et al., 2003; Casal, 2013; Pierik and de Wit, 2014; Roig-Villanova et al., 2019; Fernández-Milmanda and Ballaré, 2021; Roeber et al., 2021). Cryptochrome-mediated signal transduction is divided into different ways through interacting with various proteins of plants, for example, the CRY/COP1/SPA complexes, cryptochrome-interacting basic helix-loop-helix (CRY/CIB) complexes, CRY/PIF complexes, and so on (Jiao et al., 2007; Liu et al., 2011; Wang and Lin, 2020). Another photoreceptor that regulates the transcription factors of light signaling through targeting the COP1/SPA1 complex is UVR8 for stabilization of HY5 to initiate UVB-mediated gene expression (Cloix et al., 2012; Huang et al., 2013). On the other hand, UVR8 could directly interact with BRI1-EMS-SUPPRESSOR1 (BES1); BES1-INTERACTINGMYC-LIKE1 (BIM1) and WRKYDNA-BINDINGPROTEIN36 (WRKY36). to function in photomorphogenesis (Liang et al., 2018; Yang et al., 2018a). ZTL/FKF1/LKP2 family proteins transduce blue light signals primarily by altering the activity of the Skp1-CUL1-F-boxprotein (SCF) E3 ligase complex, which mediates the SCF E3 ligase targeted proteins degradation for circadian clock and photoperiodic flowering regulation (Song et al., 2014; Zoltowski and Imaizumi, 2014). Phototropin is also a well-known blue light photoreceptor; it has been reviewed that PHOT1 could interact with NON-PHOTOTROPIC HYPOCOTYL 3 (NPH3) and PHYTOCHROME KINASE SUBSTRATE 4 (PKS4) to elicit photomorphogenic responses (Demarsy et al., 2012). These photoreceptors sense distinguished light spectrum for orchestrating the signaling transductions of plants such as hormone signaling to regulate plant growth, development, and defense responses. The next part will focus on the plant defense responses regulated by light signaling.

## Light Regulated Plant Innate Immunity Against Insect-Borne Pathogens

The innate immune response is a well-studied defense pathway in plants. The classical defense and counter defense responses between plants and pests or pathogens are based on the “herbivore-/microbe-associated molecular patterns (HAMPs/MAMPs) to pattern-triggered immunity (PTI)” and “herbivore-/microbe-derived effectors to effector-triggered immunity (ETI)” (Zhou and Zhang, 2020; Ye et al., 2021). Relying on these two immune pathways, plants cannot only recognize but also resist insects and pathogens. Although the latest studies reveal that there may not be a clear boundary between plant PTI and ETI as the cooperation of these two pathways for promoting resistance, while the defense responses of PTI and ETI are usually considered to be different from each other (Ngou et al., 2021; Yuan et al., 2021). PTI acts as a basal immune response usually recognizes pathogens elicitors by pattern recognition receptors (PRRs) localized on the cell surface (such as cell walls and cell membranes), which triggers relatively mild defensive responses, such as ROS and nitric oxide (NO) inducement, mitogen-activated protein kinases (MAPKs) activation, phytohormones regulation, callose deposition, and pathogenesis-related (PR) genes expression, which ultimately inhibits non-adapted microbes to infect plants (Bigeard et al., 2015; Waheed et al., 2021). While ETI acts as a secondary response commonly recognizes pathogen effectors by intracellular localized resistance (R) proteins, which can produce robust defensive responses, such as hypersensitive response (HR; Balint-Kurti, 2019; Waheed et al., 2021).

Accumulating reports are complementing and perfecting the blueprint of plant innate immune pathways to fight against invaders. Higher plants have evolved a series of cell surface and intracellular immune receptors for sensing and resisting pathogen infections and herbivore infestations. Different types of PRRs have been identified including leucine-rich repeat (LRR), lysine motifs (LysMs) containing receptor proteins, and lectin-type PRRs binding extracellular ATP or bacterial lipopolysaccharides (LPSs; Ye et al., 2021). The functions of these PRRs that recognize elicitors from pathogenic organisms such as bacterial flagellin, fungal chitin, and herbivorous fatty acid–amino acid conjugates (FACs) were largely reviewed by numerous excellent articles (Boutrot and Zipfel, 2017; Saijo et al., 2018; Abdul Malik et al., 2020). R proteins functions in ETI are mainly intracellular nucleotide-binding (NB) LRR domain receptors (NLRs), which recognize specific effectors derived from pathogens or pests including genes such as *N* gene from tobacco to tobacco mosaic virus (TMV), *Sw5b* from tomato as well as *Tsw* from pepper to tomato spotted wilt orthotospovirus (TSWV; Erickson et al., 1999; Zhu et al., 2019). In consistence with the interaction model of plant-insect, plant–bacteria, and plant–fungi in the innate immune pathway, no conserved elicitor from the virus has been found, although many reports indicate that viruses can trigger PTI and ETI responses in plants and many virus-encoded proteins are considered to be the most important suppressors to counter plant defenses. Therefore, the plant defense response to the virus has its unique classical pattern such as RNAi. Actually, it is considered by researchers that RNAi works as PTI to recognize virus-derived elicitors small RNAs and is suppressed by viral effectors (Zvereva and Pooggin, 2012; Nakahara and Masuta, 2014). Cases and mechanisms involved in plant innate immune responses to fight against insect-borne pathogens by PTI and ETI have been broadly reported and summarized. An interesting study revealed a whitefly-transmitted begomovirus cotton leaf curl Multan virus (CLCuMuV), which encoded a pathogenic factor βC1 by its associated betasatellite that targeted a newly identified key factor of plant immune response pathway, WRKYDNA-BINDINGPROTEIN20 (WRKY20) transcription factor, to redeploy plant chemical immunity within the leaf for benefiting virus whitefly vectors while negatively affecting two non-vector competitors (Zhao et al., 2019). With the deepening of research, it will be found that the defense and counterdefense between plants and attackers are becoming more and more complex. These previous studies will promote us to research more complicated interaction systems and scarcely studied pathogens, which cause huge damage to agriculture. For example, Huanglongbing, the causal agent of it is citrus psyllid-transmitted *Candidatus Liberibacter asiaticus* (CLas), is an intractable plant disease that limits the yield of citrus. Due to the unculturable and phloem-restrictive characteristics, studies of CLas face great challenges (Ferrarezi et al., 2020). Therefore, exploring the elicitors and effectors from CLas and its insect vector-citrus psyllid have significant roles in finding effective strategies against Huanglongbing disease.

Tremendous reports demonstrated the essential roles of a certain spectrum of light in promoting plant defense against pathogen infection and herbivore infestation. Normally, red light increases plant resistance to various pathogens, herbivores, and nematodes (Yang et al., 2018b; Gallé et al., 2021), although the molecular mechanisms were still obscure. As described above, plant innate immunity is an essential strategy deployed by plants to counter invaders. The light could regulate plant resistance to pathogens through manipulating the plant innate immunity pathway, especially for the *R* gene regulated resistance. Wu and Yang (2010) showed that blue light photoreceptor CRY1 is involved in promoting R protein-mediated plant resistance to *Pseudomonas syringae* pv. tomato DC3000 carrying avrRpt2 in *Arabidopsis*. The effector-triggered local resistance and systemic acquired resistance (SAR) were both impaired in the *cry* mutant and salicylic acid (SA)-induced *PR* gene *PR-1* expression is reduced as well. Despite the light-mediated *R* gene resistance to bacteria, a similar agent also occurred in an insect-borne virus. Chandra-Shekara et al. (2006) demonstrated that light was required for R protein hypersensitive response to TCV (HRT)-mediated HR and resistance to turnip crinkle virus (TCV). Jeong et al. (2010) further showed that blue-light photoreceptors, CRY2 and PHOT2, maintained post-transcriptional stability of HRT, thereby resisted TCV. These studies unrevealed the resistance regulated functions of essential factors in light signaling, which provides a significant insight into the further exploration of light signaling-mediated plant defense against insect-borne pathogens.

## Light-Mediated Phytohormone Signaling Against Insect-Borne Pathogens

Hormone signaling pathways regulate plant growth, development, and defense responses in their whole life. Among the various phytohormones, SA, jasmonic acid (JA), and ethylene (ET) are widely reported to be resistant to biotic stresses. Generally speaking, SA is usually considered as a primary hormone against biotrophic, hemibiotrophic pathogens, and phloem-feeding insects; JA and ET mainly regulate plant immune responses against chewing insects and necrotrophic pathogens (Lazebnik et al., 2014; Li et al., 2019; Gallé et al., 2021). Understanding how plants coordinate phytohormone signaling to counteract serious external threats makes great sense for exploring novel disease resistance strategies.

Jasmonic acid signaling is initiated by the generation of jasmonoyl-L-isoleucine (JA-Ile), once plants perceive stimuli from the external environment. Then, JA-Ile binds to F-box protein CORONATINE INSENSITIVE1 (COI1) to degrade JAZ through the 26S proteasome pathway for releasing JASMONATE ZIM-DOMAIN PROTEIN (JAZ) inhibited downstream genes expression of JA signaling such as transcription factor (TF) families of MYCs, MYBs, WRKYs, etc., (Ruan et al., 2019; Wu and Ye, 2020). These TFs regulate plant defense responses to pathogens and herbivores by activating the resistance genes expression. For instance, *terpene synthase* (*TPS*) genes for synthetizing TPS, whose products could regulate herbivore appealing or avoiding (Tholl, 2015; Chen et al., 2020). *Vegetative storage proteins (VSPs)* genes encoded toxic proteins mainly induce defensive responses to fight against insect herbivores (Schweizer et al., 2013; Wu and Ye, 2020). Multiple articles have demonstrated the resistance roles of JA signaling in plant-pathogen interactions, especially when concerned with the complicated tripartite interactions, which usually occur when vector-borne pathogens infect plants. For example, begomovirus tomato yellow leaf curl China virus (TYLCCNV)-associated betasatellite encoded βC1 hijacks the core factors, such as JA signaling pathway MYC2 and light signaling pathway PIFs, to repress their transcription factor activities, which mediates *TPS* gene expression for promoting the attraction of virus vector-whitefly (Li et al., 2014; Zhao et al., 2021). Except for begomoviruses, which belong to the DNA virus, the vector-borne RNA virus was also reported to repress JA signaling for promoting their vector performance. TSWV, which is transmitted by thrips, encodes a nonstructural-protein (NSs) for interacting with and disturbing the function of MYC2/3/4 to disable JA-mediated activation of *TPS* genes. This modifies host volatiles and increases vector preference, which ultimately promotes vector performance in the host (Wu et al., 2019).

**FIGURE 1:**
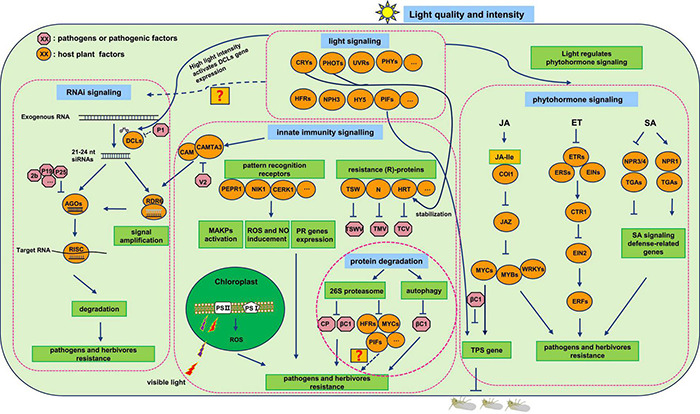
Light-regulated plant defense pathways.

Salicylic acid signaling has profound importance in regulating plant resistance against biotrophic, hemibiotrophic pathogens, and some phloem-feeding herbivores, which usually cause minor damage to plants (Onkokesung et al., 2016; Li et al., 2021). Two classes of receptors, NONEXPRESSER OF PR GENES1 (NPR1) and NPR3/NPR4, perceive SA, although they play opposite roles in regulating defense gene expression (Figure 1; Zhang and Li, 2019; Zhou and Zhang, 2020). NPR1, as the transcription activator in SA signaling, functions in sensing SA and repressing the transcriptional inhibition activities of NPR3/NPR4, which promotes the expression of defense-related genes, *PR* genes (Liu et al., 2015; Ding et al., 2018). SA plays a significant role in plant SAR, which is considered as an essential pathway for disease resistance. A growing number of researchers have reported the SA signaling-mediated plant resistance to insect-borne pathogens, which will fulfill our understanding of plant disease defense strategies and promote the process of exploration of disease-resistant cultivars.

Ethylene is well-known for regulating plant growth, fruit ripening, and stimulation of seed germination. Although ET signaling also plays a pivotal role in plant defense responses, the related cases are not widely reported as for JA and SA signaling. ET signaling initiates through ET perceiving by membrane-localized receptor dimer protein kinases such as ETHYLENE RESPONSE1/2 (ETR1/ETR2), ETHYLENE RESPONSE SENSOR1/2 (ERS1/ERS2), and ETHYLENE INSENSITIVE4 (EIN4), which releases the inhibition of these receptors to ET signaling followed by a series of protein activation and degradation processes, and then ET signaling-responsive genes express to regulate plant growth and resistance (Figure 1; Ju and Chang, 2015; Waadt, 2020). The TFs of the ethylene response factor (ERF) family are found to regulate plant resistance to a variety of pathogens and insects. For example, ERF3 identified in rice (*Oryza sativa*) positively affects gene expression of trypsin proteinase inhibitors as well as mediates resistance toward *Chilo suppressalis* caterpillars (Lu et al., 2011). This study provides a novel breeding target for plants to resist insects as well as their transmitted pathogens.

Light-mediated phytohormone signaling plays significant roles in regulating plant defense responses, as it was proved by various articles that a series of monochromatic light, such as red light, blue light, and UV light, displayed plant defense enhancement functions by activating phytohormone signaling pathways (Ballaré, 2014). Moreover, it is generally accounted for the red light, some reports also recorded that blue light and UV light could enhance plants’ SA and JA signaling, whereas FR or low R:FR ratio compromised these signaling pathways (Ballaré, 2014). For instance, red light illumination overnight enhanced host resistance against *Pseudomonas syringae pv. tomato* DC3000 (*Pto* DC3000), as this treatment elicited SA accumulation and the expression of defense-related genes in tomato (*Solanum lycopersicum* L.) plant leaves (Yang et al., 2015). Despite the resistance to bacterial pathogens, red light also contributes to defending against fungi such as broad beans (*Vicia faba* L.) infecting *Botrytis cinerea*, rice infecting *Magnaporthe grisea*, and cucumber infecting *Sphaerotheca fuliginea*. The resistance pathways activated by a red light to defend these fungi are *via* influencing hydrogen peroxide (H_2_O_2_), ascorbate peroxidase (APX), and catalase (CAT) enzyme activities (Ueno et al., 2007; Wang et al., 2010; Ahn et al., 2015). Moreover, red light also induces systemic resistance to fight against root-knot nematode by coordinating regulation of SA, JA, and redox signaling in watermelon (Yang et al., 2018b). Red light-mediated defense response to fight against the insect-borne virus through phytohormone signaling was also reported. In this case, SA levels and SA-mediated *PR-1*, *PR-2*, and *PR-5* expression in *Nicotiana tabacum* (*N. tabacum*) were increased by red light treatment and these responses effectively delayed symptom expression and replication of cucumber mosaic virus (CMV) on *N. tabacum*, notably blue light treatment made the same effect as red light treatment in this study (Chen et al., 2015). In addition to CMV, TMV was also reported to be defended by the host through UV light eliciting SA signaling (Yalpani et al., 1994). Ðinh et al. (2013) have reported that UVB increased phytohormones accumulation of JA, JA-Ile, and abscisic acid (ABA), which play important roles in regulating plant defense against the biotic and abiotic stresses. Despite these lights eliciting phytohormone-regulated resistance, FR or low R:FR ratio was generally commended as a negative regulator for increasing phytohormone-regulated responses and tremendous studies have illustrated the attenuated SA- and JA-dependent defense responses against various attackers from pathogens to herbivores (Table 1). These studies describe the essential functions of light with specific wavelength in regulating plant hormone pathways to resist pathogens, though the detailed molecular mechanisms are unclear, especially the essential host plant regulators involved and how pathogens or insects evolved to escape host defense.

**TABLE 1:** Light-mediated plant resistance to pathogens and herbivores.

Pathogen or herbivore type		Host plants	Light	Photo-receptors	Mechanisms	References
Viruses	tobacco mosaic virus	*Nicotiana tabacum*	UV-C		Increasing SA, PR1a and PR1b activation	Yalpani et al., 1994
	soybean mosaic virus	*Glycine max*	R:FR ratio = 5.92		Plant defense-related genes were upregulated under normal light compared with shade	Zhang et al., 2019
	cucumber mosaic virus	*Nicotiana tabacum*	Blue and red		Increasing SA levels and SA-mediated *PR-1*, *PR-2* and *PR-5* expression activation	Chen et al., 2015
	turnip crinkle virus	*Arabidopsis*	Blue	CRY2 and PHOT2, positive	Maintaining post-transcriptional stability of R protein (HRT) and promoting HRT-mediated HR for TCV resistance.	Chandra-Shekara et al., 2006; Jeong et al., 2010
Herbivores	*Spodoptera litura*	*Arabidopsis*, *Nicotiana tabacum*, *Oryza sativa*, and Zea may	UV-B		Eliciting JA-regulated glucosinolates (GSs), and an unidentified anti-insect metabolite(s)	Qi et al., 2018
	*Mamestra brassicae*	*Solanum lycopersicum*		phyB1 and phyB2, positive	Maintaining constitutive (densities of glandular trichomes) as well as direct and indirect defenses which induced by methyl jasmonate (MeJA).	Cortés et al., 2016
	*Bemisia tabaci*	*Cucumis sativus*	R:FR ratio = 1.2		Probably due to lower chlorophyll content and thinner leaves	Shibuya et al., 2010
	*Pieris brassicae*	*Arabidopsis*	low R: FR		Suppressing methyl-jasmonate-induced volatiles and terpenoids	Kegge et al., 2013
	*Nezara viridula Piezodorus guildinii*	*Glycine max*	UV-B		Increasing isoflavonoids daidzin and genistin	Zavala et al., 2015
	*Tupiocors notatus*	*Nicotiana attenuata*	UV-B		Increasing the accumulation of 17-hydroxygeranyllinalool diterpene glycosides and defensive proteinase inhibitor proteins	Ðinh et al., 2013
	*Spodoptera littoralis*	*Arabidopsis*	Low R: FR		Low R: FR causes the upregulation of sulphotransferase (ST2a) a phyB/PIF-dependent manner, which is responsible for the reduction of the active JA pool	Fernández-Milmanda et al., 2020
		*Phaseolus lunatus*	High R: FR		Increasing the secretion of extrafloral nectar, which is activated by JA and functions as an indirect defense mechanism against herbivores.	Radhika et al., 2010
	*Manduca sexta*	*Nicotiana longiflora*	FR		Suppress the expression of several defense-related genes and inhibiting the accumulation of herbivore-induced phenolic compounds.	Izaguirre et al., 2006
	*Frankliniella occidentalis*	*Solanum lycopersicum*	UV		Probably activating of JA-associated signaling, but not plant secondary metabolism or trichome-related traits.	Escobar-Bravo et al., 2019
Bacteria	*Pseudomonas syringae pv. tomato DC3000*	*Arabidopsis*		CRY1, positive	Enhancing both local resistance and systemic acquired resistance	Wu and Yang, 2010
		*Solanum lycopersicum*	Red		Eliciting SA accumulation and the expression of defense-related genes	Yang et al., 2015
		*Arabidopsis*	Low R: FR	phyB, positive	Compromising both SA- and JA-dependent pathogen defenses	de Wit et al., 2013
Fungi	*Botrytis cinerea*	*Arabidopsis*	Low R: FR	phyB, positive	Decreasing the expression of defense markers (*ERF1* and *PDF1.2*) induced by *Botrytis cinerea via* a SA-independent mechanism that requires the JAZ10 transcriptional repressor	Cerrudo et al., 2012
				phyB, positive	Compromising both SA- and JA-dependent pathogen defenses	de Wit et al., 2013
					Reducing the biosynthesis of indolic glucosinolates and camalexin	Cargnel et al., 2014
	*Fusarium oxysporum*	*Arabidopsis*		phyB, positive	Promoting JA-dependent defenses	Kazan and Manners, 2011
	*Sphaerotheca fuliginea*	*Cucumis sativus*	Red		Maintaining higher levels of H_2_O_2_ and SA, and stronger expression of defense genes such as *PR-1*.	Wang et al., 2010
	*Botrytis cinerea*		Blue and red		Promoting the accumulation of stilbenic compounds and differential expression of genes involved in defense response	Ahn et al., 2015

*SA, salicylic acid; JA, jasmonic acid; FR, far-red.*

Genetic experiments confirmed the important roles of photoreceptors in light-mediated resistance responses with indication *phyB* mutants of various species of plants showing increased sensitivity to herbivores and pathogens (Cortés et al., 2016; Courbier et al., 2020). These studies demonstrated that red light-mediated plant defense response was initiated along with the perception of red light by photoreceptor phyB and then phyB primed downstream resistance responses. Actually, phyB inactivation destabilizes MYC stability in a COP1-dependent manner (Chico et al., 2014). In addition, inactivating phyB causes more available JAZ10, a negative regulator of JA signaling, which attenuates JA signaling-mediated defense responses (Leone et al., 2014). These studies indicated that red light regulated protein stabilities of plant hormone pathways through its photoreceptor to orchestrate plant defense responses to fight against attackers. While red light enhanced plant defense is not suitable for all the interaction cases between plants and pathogens, especially when concerned with multiple interactions and specific pathogens. A recent study about red light-mediated tripartite interaction of plant-begomovirus-whitefly demonstrated the beneficial effects of red light on both the whitefly and the begomovirus. Zhao et al. (2021) found that red light promoted the mutualism of whitefly-begomovirus by stabilizing βC1 protein encoded by TYLCCNV-associated betasatellite and accumulated βC1 further inhibits PIFs positively controlling of plant defenses against whitefly by reducing the promoter-binding activity of PIFs to *TPS* genes. Furthermore, βC1 also decreased the transcriptional activity of PIFs and MYC2 *via* disturbing their dimerization, thus impairing plant defenses against TYLCCNV transmitted vector-whitefly.

## Light-Mediated RNA Interference Signaling Pathway Against Insect-Borne Pathogens

Ribonucleic acid interference (RNAi) (also called RNA silencing) is involved in broad regulative pathways with nucleotide sequence specific, and it is mediated by small RNAs. The small RNAs include microRNAs (miRNAs), short-interfering RNAs (siRNAs), PIWI-related RNAs (piRNAs), and so on. Unlike piRNAs, which are only found in animals, miRNAs and siRNAs are found in most eukaryotes and function as the primary factors for guiding the antiviral immune process in plants (Martínez de Alba et al., 2013; Muhammad et al., 2019; Niehl and Heinlein, 2019). In addition to the three types of small RNAs for priming RNAi, three core families of proteins for achieving RNAi-involved defense are indispensable equally such as Dicer-like (DCL) protein, RNA-dependent RNA polymerase (RDR), and argonaute (AGO) protein. These proteins coordinate together to fine-tune plant resistance to pathogens and herbivores. As a counterdefense strategy, pathogens employ their multifunctional proteins as suppressors to defend against RNAi by targeting the essential proteins in this pathway. So far, large numbers of viral suppressors of RNAi (VSRs) have been characterized from all the plant virus families. The main antagonize mechanism employed by these VSRs is to interfere with the various steps of the RNAi pathway (Li and Ding, 2006; Song et al., 2011). For example, VSRs of diverse plant viruses suppress siRNA production, siRNA sequestration, and systemic silencing. The typically representative VSRs are Potyvirus helper component-proteinase (HC-Pro), cymbidium ringspot virus (CymRSV) P19, and potato virus X (PVX) P25, respectively (Voinnet et al., 2000; Mallory et al., 2002; Lakatos et al., 2004). The development of protein-protein interaction experiments has identified many key players of the plant RNAi pathway targeted by VSRs as an important strategy for viral anti-RNAi. In a number of studies, VSRs (TCV CP, CMV 2b, tombusvirus P19, PVX P25, polerovirus P0, and P1 of sweet potato mild mottle virus) have been identified that could target AGO1 for its degradation or interfering its function (Zhang et al., 2006; Bortolamiol et al., 2007; Azevedo et al., 2010; Chiu et al., 2010; Giner et al., 2010; Várallyay et al., 2010; Derrien et al., 2012). Except for AGO1, RNAi pathway key proteins of DCLs and other AGOs were also reported to be attacked by VSRs for disturbing their function (Lacombe et al., 2010; Hamera et al., 2012; Ramesh et al., 2014; Csorba et al., 2015). As an antivirus pathway initiated by viral-derived siRNAs, RNAi also defends against DNA viruses (Bisaro, 2006). It was reported that DICER-like 3 (DCL3) plays an important role in resistance against DNA viruses and presumably *via* DNA methylation (Akbergenov et al., 2006; Blevins et al., 2006; Raja et al., 2014). Several DNA virus-encoded VSRs have been identified to counteract RNAi including ACMV AC4 and AC2, CaLCuV AL2/AC2, TYLCCNV βC1, etc., (Chellappan et al., 2005; Cui et al., 2005; Buchmann et al., 2009). Except for directly targeting the RNAi pathway of virus VSRs, it has been reported recently that virus-encoded protein (CLCuMuV V2) could interfere with the interaction of CaM-CAMTA3 in calcium signaling, which positively regulates the gene transcription of RNAi key components RNA-dependent RNA polymerase 6 (RDR6) and Bifunctional nuclease-2 (BN2) to suppress RNAi (Wang et al., 2021b). These defense and counterdefense arm race between the plant’s defense machinery and viral VSRs are results of long co-evolutionary history and display a complex and sophisticated network.

Research progress about light-mediated RNAi signaling pathways is rare compared with those regulated by the phytohormone pathway. Most of these few articles focus on describing the effect of light intensity on the RNAi pathway (Kotakis et al., 2010, 2011; Patil and Fauquet, 2015). Some studies documented “high-light (HL)” intensity (130 ± 20 μmol m^–2^ s^–1^) positively affects the frequency of spontaneous post-transcriptional gene silencing (PTGS) in transgenic plants than “low-light (LL)” intensity (35 ± 15 μmol m^–2^ s^–1^) conditions (Kotakis et al., 2010). Furthermore, HL activates higher expression levels of *DCL3* and *DCL4* than that of LL, which further emphasized the regulation of light on RNAi (Kotakis et al., 2011). However, there is also some inconsistent report indicating not all the HL intensity is always good for promoting plant RNAi response. A too HL intensity (≥450 μE/m^2^/s) even confers a negative impact on the systemic movement of the silencing signal in transient agroinfiltration studies in *N. benthamiana*, whereas the viral symptom severity was reduced in this context. This phenomenon could be explained by a change in the plant sink-source relationship, which finally affected the systemic translocation of either small RNAs or the viral genome *via* the phloem (Patil and Fauquet, 2015).

The detailed mechanism of how light regulates the RNAi defense pathway is still elusive. Several future study areas could be explored to answer this important scientific question including what intensity or which spectrum of light can maximize the activation of plant RNAi pathways and what are the host factor(s) and mechanism(s) of light-regulated RNAi pathways?

## Light-Mediated Protein Stability and Defense Responses

The protein stability regulation plays a central role in plant defensive responses against the invasion of pathogens including bacteria, fungi, and viruses. The 26S proteasome pathway commonly indicates the ubiquitin 26S proteasome degradation system (UPS) in which the degraded protein needs to be ubiquitinated by a series of ubiquitin-related enzymes, namely, ubiquitin-activating enzyme (E1), ubiquitin-conjugating enzyme (E2), and ubiquitin ligase enzyme (E3) (Dielen et al., 2010). The polyubiquitinated target proteins were then loaded into 26S proteasome for their degradation. Plenty of articles have shown that the plants deploy UPS for disease resistance. Likewise, proteins encoded by several begomoviruses are direct targets of UPS degradation system. *Begomovirus* is the biggest plant virus genus consisting of more than 320 species and infects dicotyledonous plants. Most of them are the most destructive plant viral pathogens. Tomato yellow leaf curl virus (TYLCV), the coat protein (CP) encoded by its genome, is a target of UPS digestion (Gorovits et al., 2014). In addition, a well-known pathogenicity determinant βC1 encoded by TYLCCNV-associated betasatellite is found to be degraded by UPS as well (Shen et al., 2016). Besides viruses, other types of pathogens such as bacteria and fungi and their major pathogenic factors are also targeted by UPS. A recent study revealed a new ubiquitin-independent protein degradation pathway deployed by insect-vectored plant pathogenic phytoplasmas. This study demonstrated that SAP05 protein effectors from phytoplasmas hijacked the plant ubiquitin receptor RPN10 in a way that is independent of substrate ubiquitination, then promoted the concurrent degradation of two plant TFs, namely, SPL and GATA. This bacterial hijack of plant developmental regulators prolonged the host lifespan and induced witches’ broom-like proliferations of leaf and sterile shoots and ultimately facilitated parasitism of phytoplasma (Huang et al., 2021).

Autophagy has long been known as a conserved vacuole-/lysosome-mediated degradation pathway for clearing and recycling cellular components. Growing evidence has linked autophagy to immunity against invading pathogens, which elucidates the disease resistance roles of autophagy in plants. Autophagy-mediated plant defense responses are largely reported to be resistant to insect-borne viruses (Hofius et al., 2017; Ismayil et al., 2020; Yang et al., 2020). For instance, the key autophagy protein encoded by autophagy-related gene 8 (*ATG8*), which was reported to interact with βC1 encoded by CLCuMuV-associated betasatellite for degradation. Silencing of other essential proteins in the autophagy pathway, ATG5 and ATG7, reduced the resistance of the plant to a large number of DNA viruses, such as CLCuMuV, TYLCV, and TYLCCNV, which elucidated the significant roles of autophagy in plant defense (Haxim et al., 2017).

Studies about light signaling-mediating 26S proteasome and autophagy pathway to fight against attackers are rarely reported. Several well-known regulators functioning in promoting protein degradation in light signaling could provide examples for our understanding of this layer of light regulation on disease resistance. For example, phytochromes, such as phyA and phyB, mediate the degradation of PIFs *via* the 26S proteasome pathway for light response regulation (Park et al., 2018). Otherwise, the positive and negative factors of photomorphogenesis, PIF1 and HFR1, undergo reciprocal co-degradation *via* the 26S proteasome pathway in the dark to optimize photomorphogenesis (Xu et al., 2017). These studies illustrated the important roles of core factors in light signaling in protein accumulation regulation, which is possibly also essential for regulating the protein functions in other plant defense pathways. Actually, there are several vital regulators in plant resistance pathways, which are reported to be sensitive to light, such as ETHYLENE RESPONSE FACTOR1 (ERF1), a crucial factor in the biotic and abiotic stress responses, were reported to be unstable in the dark (Cheng et al., 2017). Other key factors of JA defense response pathway, MYCs and JAZs, their protein accumulations were also regulated by different light or dark conditions *via* 26S proteasome, and these processes were phytochromes dependent (Chico et al., 2014). These studies indicated the significant roles of light signaling in regulating protein degradation. Meanwhile, whether light signaling regulators could interact with proteins in the 26S proteasome or autophagy pathway, such as RPNs and autophagy-related proteins (ATGs), they wait for further exploring for understanding the detailed defensive response mechanisms regulated by light.

## Light-Engineering Technology for Insect Vector Controlling

A key player in vector-borne pathogen transmission is the insect vectors, which facilitate pathogens to transmit among hosts. Therefore, inhibiting the population of vector insects or reducing their fitness on plants plays an important role in the prevention and control of vector-borne diseases. Due to the destructive effects of pesticides on the environment, biological and physical strategies for insect control will be very helpful for sustainable agriculture. Rapidly developed greenhouse agriculture has tried to supplement LED lighting systems to optimize crop production and quality, which provides a breakthrough and profound prospect for pest and disease controlling in a light-engineering technology (Gallé et al., 2021; Lazzarin et al., 2021). Therefore, it makes great sense for exploring the effects and mechanisms of light-mediated resistance to fight against herbivores. The effects of light on herbivores’ behavior consist of direct and indirect regulation. As the insects’ compound eyes contain visual pigments, which enable them to perceive different wavelengths of light. Therefore, revealing the mechanism by which light affects insect behaviors makes great sense for developing environmentally friendly insect control strategies. A recent study has elucidated the clock genes, temperature, and light affecting mosquito mating, it might lead to novel vector control strategies based on the light treatment that target insect reproductive behavior (Wang et al., 2021c). Similar to this, plant insect pests also have a visual system and it was reported that whiteflies were more attracted by a 550-nm wavelength of green LED and a 469-nm wavelength of the blue LED was proved to be most inhibitory (Stukenberg and Poehling, 2019). However, it might be more complicated when considering pest control through light in agriculture, as the indirect influence of light-mediated plant resistance to insect pests plays an important role. Therefore, uncovering the mechanisms of light signaling-regulated plant defense to herbivores has great significance in greenhouse agriculture. In the following part, we will review the indirect regulation of light on herbivores, which focuses on targeting different plant resistance pathways.

A number of studies have documented a series of the specific spectrum of light that could induce various plant defense responses to herbivores by distinguished mechanisms. For instance, UVB light enhances the resistance of multiple plant species, such as *Arabidopsis*, tobacco, rice, and maize to *Spodoptera litura*. The mechanism is through eliciting the JA-regulated glucosinolates (GSs) and an unidentified anti-insect metabolite(s) (Qi et al., 2018). Kegge et al. (2013) showed that low R:FR ratio and severe shading conditions suppressed both the constitutive and methyl jasmonate-induced volatiles and terpenoids in *Arabidopsis*, and volatile organic compound (VOC)-based preference of *Pieris brassicae* caterpillars was significantly affected by the R:FR ratio. Despite influencing anti-insect metabolites and volatiles of plants, other studies, which elucidated a distinguished mechanism of light-regulated plant defense to insects, are based on changing plant morphology. For example, cucumber seedlings treated by fluorescent lamps (FLs) (R:FR ratio was 7.0) were less attractive to whitefly than that of metal-halide lamps (MLs) (R:FR ratio was 1.2), which were considered probably due to changes in morphologic characteristics such as the leaf color and thickness resulting from high R:FR illumination of FL (Shibuya et al., 2010). Furthermore, *phyB1phyB2* double mutant tomato showed reduced densities of glandular trichomes (Cortés et al., 2016). Escobar-Bravo et al. (2017) summarized the herbivore defense functions by UVB light treatment in different plants and insect interaction systems. Except for these light-regulated plants’ direct resistance to herbivores, a few studies demonstrated light-mediated plant indirect defense against herbivores, which was achieved by promoting host attractions to insect predators. For example, high R:FR ratio induced JA-controlled extrafloral nectar (ER) secretion of lima bean (*Phaseolus lunatus*). ER is considered to be activated by JA and functions as an indirect defense mechanism against herbivores (Radhika et al., 2010). Furthermore, inactivation of phyB regulated signaling, which mutated the two *phyB* genes in tomato or treated tomato plants with a low R:FR ratio compromised both the direct and indirect defenses, which induced by methyl jasmonate (MeJA). The result showed that predatory mirid bug (*Macrolophus pygmaeus*) preferred VOCs from plants in which *phyB* was inactivated over VOCs from the control plants (Cortés et al., 2016). All these studies elucidated the significant roles of light pathway in regulating plant resistance to herbivores.

## Prospects of Light-Engineering Technology for Enhanced Plant Disease Resistance

The outspreading of tremendous emergence and reemergence of plant diseases are mostly caused by vector insects, which accelerate the transmission of pathogens among hosts (Gao et al., 2010; Heck and Brault, 2018; Wang et al., 2019; Chiapello et al., 2021). Therefore, researching for efficient strategies to prevent and control insect-borne diseases is of great significance for ensuring the safety of food production. The mechanism of light-mediated plant resistance will provide new directions for developing plant disease control strategies based on light treatment and crop breeding. From the studies reported previously, it could be concluded that most studies illustrated red light as a positive regulator for plant defense response and FR light as a negative factor. Most of the literature has demonstrated the impaired resistance of plants to pathogens under FR light treatment (Cerrudo et al., 2012; de Wit et al., 2013; Courbier et al., 2020, 2021). That explains why the red spectrum of LED lighting systems is usually used in greenhouses for crop disease management (Gallé et al., 2021; Lazzarin et al., 2021). According to the resistant functions of different spectrums of light to distinguish pathogens and herbivores, we can design optimized combinations of specific wavelength and intensity of light to prevent and control specific types of plant diseases. Based on the studies on the host resistance responses of different light treatments to cure various plant diseases, the environmental light quality and intensity could be tailed for light engineering with LEDs in an individual greenhouse for precise disease control. Of course, with the assistance of artificial intelligence technology, it is also possible to balance crop yield and disease resistance by light-engineering technology. We can setup an intelligent light control system to satisfy different requirements of plants (disease resistance or growth), which provides the optimal growth conditions at each growth stage of the plants. Furthermore, we can also use different lights to prevent and control different plant diseases. These ideas about disease prevention and control in green agriculture are all based on the mechanism revelation of plant defense to different pathogens or herbivores by specific light treatment.

Besides understanding specific wavelength of light-mediated plant resistance to pathogens, the exploration of molecular mechanisms is also pivotal when considering applied purposes in breeding disease-resistant crops. Other studies could also explore the mechanisms of how light signaling regulates plant resistance. For example, which photoreceptor(s) regulate the biosynthesis of terpenoid and other VOCs, which function as communication signals with the host plant community together with various insects, e.g., vector, non-vector, predator, and parasitoids? How does light signaling intercross with the RNAi pathway? The rapid development of gene editing technology can enable us to obtain crops with both disease resistance and high-yielding traits.

## Conclusion

Exploring novel resistance pathways in plants is of great significance for enhancing the broad-spectrum disease resistance of crops. In this review, we propose a new idea about light-engineering technology combining both the optimal external LED-based environmental conditions and optimal plant internal disease gene networks. The final aim is to provide enhanced disease resistance and high yield crop performance. Tailed light conditions confer better plant resistance against insect-borne pathogens and insect vectors. Exploring how to use light to control insect-borne diseases will provide broad prospects for the development of green agriculture. We believe that light-engineering technology, which is combined with big data technology and LED technology, will provide human beings with a high yield of crops and also safer food in the future.

## Author Contributions

JY and DW conceived this manuscript. DW performed the literature search and drafted the manuscript. JY, JQ, and BD revised the manuscript. All authors contributed to the article and approved the submitted version of the manuscript.

## Conflict of Interest

The authors declare that the research was conducted in the absence of any commercial or financial relationships that could be construed as a potential conflict of interest.

## Publisher’s Note

All claims expressed in this article are solely those of the authors and do not necessarily represent those of their affiliated organizations, or those of the publisher, the editors and the reviewers. Any product that may be evaluated in this article, or claim that may be made by its manufacturer, is not guaranteed or endorsed by the publisher.
